# The Implementation and Clinical Validation of a Whole-Genome Sequencing Non-Invasive Prenatal Test in a High-Complexity Clinical Laboratory: A Retrospective Analysis of 11,235 Pregnancies

**DOI:** 10.3390/diagnostics16182936

**Published:** 2026-09-11

**Authors:** Lesley E. Northrop, Jay Eastway, Sudhakar Ravuri, Genevieve Fairbrother

**Affiliations:** 1 Atlanta Women’s Health Group Laboratory, Alpharetta, GA 30022, USA; 2 Atlanta Women’s Health Group, Alpharetta, GA 30022, USA; gfairbrother@awhg.org

**Keywords:** non-invasive prenatal testing, NIPT, cell-free DNA, whole-genome sequencing, aneuploidy screening, trisomy 21, positive predictive value, clinical laboratory, VeriSeq

## Abstract

**Background**: Cell-free DNA non-invasive prenatal testing (NIPT) is recommended by the American College of Obstetricians and Gynecologists (ACOG) and the American College of Medical Genetics and Genomics (ACMG) for fetal aneuploidy screening in all pregnancies, yet most testing is performed by centralized reference laboratories. We evaluated whether a whole-genome sequencing (WGS) NIPT assay implemented in a high-complexity clinical laboratory can achieve a performance consistent with established reference laboratories. **Methods**: We retrospectively analyzed 11,235 evaluable clinical NIPT samples processed in a single Clinical Laboratory Improvement Amendments (CLIA)-certified, College of American Pathologists (CAP)-accredited laboratory using the Illumina VeriSeq NIPT Solution v2, which reports trisomy 21 (T21), trisomy 18 (T18), trisomy 13 (T13), and sex chromosome aneuploidies (SCA). Results were compared with the diagnostic and clinical follow-up; the performance was calculated overall and by maternal age with 95% confidence intervals (CIs). **Results**: Of 11,318 accessioned samples, 83 (0.73%) were not reportable and excluded. The sensitivity was 100% for all conditions (T21 95% CI 92.1–100%); per-condition specificity exceeded 99.9% (99.81% for any reported aneuploidy overall), with no false-negative results among cases with available follow-ups. Observed positive predictive values (PPVs) were 91.8% (T21), 85.7% (T18), 63.6% (T13), and 79.3% (SCA), consistent with published ranges for this routine, unselected screening population. **Conclusions**: A high-complexity clinical laboratory can deliver WGS-based NIPT at this level of performance, supporting broader, more timely access to guideline-recommended screening. NIPT remains a screening test, and high-risk results require diagnostic confirmation.

## 1. Introduction

The clinical significance of NIPT lies in its superior screening performance relative to traditional serum biochemistry and ultrasound-based screening. Screening tests identify individuals in a population who have a high likelihood of being affected by a condition; they are typically non-invasive and carry minimal direct physical risk to the patient. By analyzing cell-free DNA (cfDNA) circulating in maternal plasma, NIPT provides a highly accurate, non-invasive method for assessing the risk of common chromosomal aneuploidies. cfDNA screening is, however, more costly than traditional serum screening and is not uniformly covered by insurance; for uninsured or underinsured patients, the out-of-pocket cost can be a barrier to access.

Professional organizations, including the American College of Obstetricians and Gynecologists (ACOG) and the American College of Medical Genetics and Genomics (ACMG), recommend NIPT as a primary screening option for all pregnant patients regardless of the baseline risk [1,2]. These recommendations reflect the superior accuracy of NIPT in detecting Down syndrome compared with traditional first- or second-trimester screening. NIPT significantly reduces false-positive rates, thereby minimizing unnecessary invasive diagnostic procedures—such as amniocentesis or chorionic villus sampling (CVS)—which carry a small risk of pregnancy loss, while at the same time increasing the detection of affected pregnancies [3].

While NIPT is now a first-line screening option, most testing has historically been referred to centralized commercial laboratories, which can introduce logistical delays between specimen collection, result reporting, and clinical counseling. Implementing a validated NIPT assay within an accredited local clinical laboratory allows the screening, interpretation, and follow-up to be integrated within the patient’s own care team, supporting integrated care, in which results are interpreted directly within the context of the patient’s clinical profile, maternal age, and ultrasound findings; timely results and counseling, in which shorter and more predictable turnaround times reduce the interval to genetic counseling and, when indicated, diagnostic testing; and clinical excellence, in which state-of-the-art next-generation sequencing (NGS) infrastructure supports medical-grade testing comparable to global reference laboratories.

This retrospective study of 11,235 pregnancies evaluates whether a high-complexity clinical laboratory can maintain high sensitivity for common aneuploidies while achieving positive predictive values comparable to those of established reference laboratories.

## 2. Materials and Methods

### 2.1. Patient Samples and Ethics

**Figure 1:**
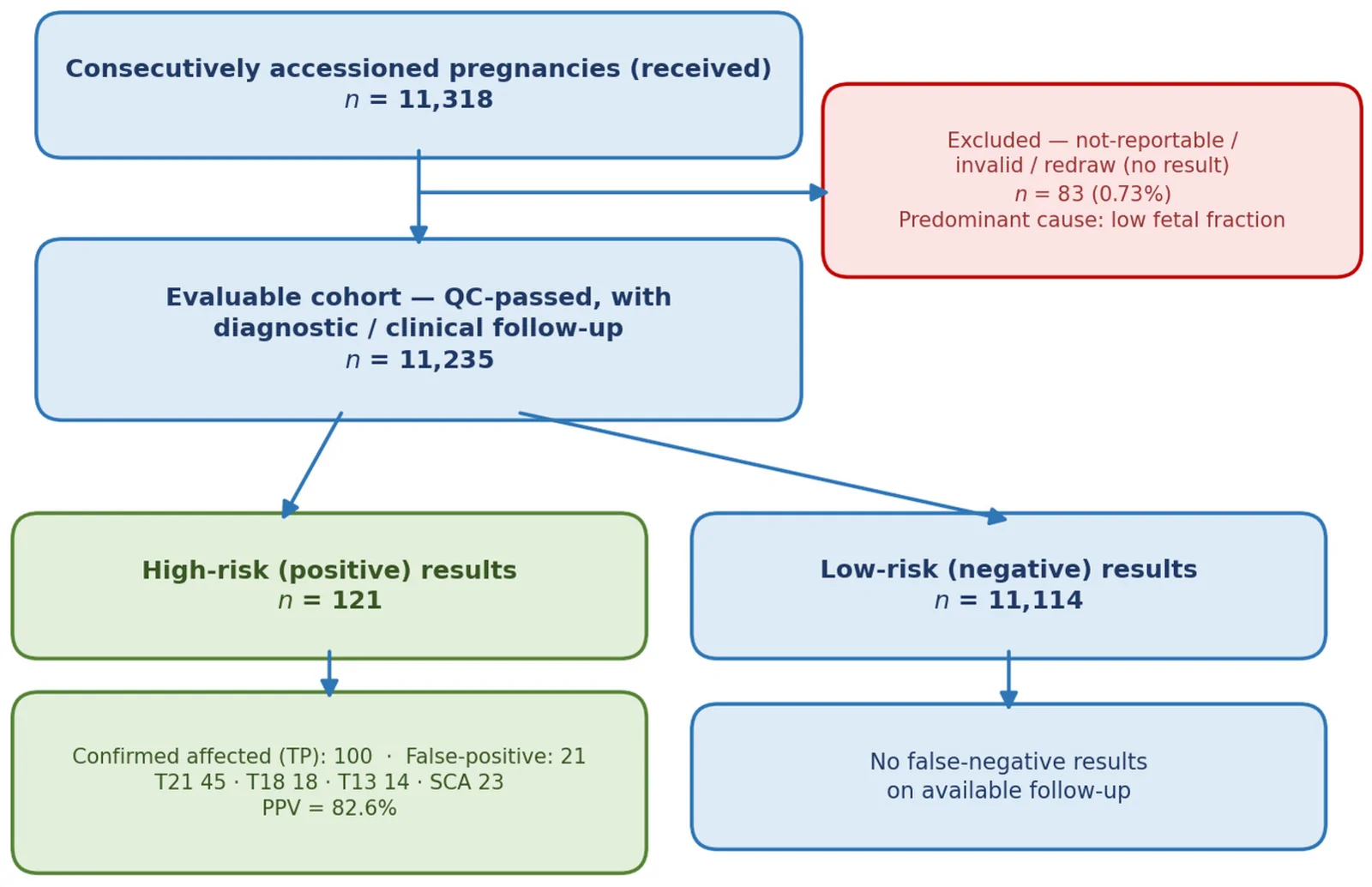
Sample flow through the study. Reported conditions were T21, T18, T13, and SCA; other chromosomal findings were outside the reporting scope. Box colours indicate node type: blue, cohort counts and low-risk (screen-negative) results; red, excluded (not-reportable) samples; green, high-risk (screen-positive) results and their confirmation.

Samples were accessioned at a private, physician-owned high-complexity clinical laboratory serving affiliated obstetrics and gynecology practices across the metropolitan Atlanta, Georgia, region; all specimens were referred from within this network of practices. Consistent with contemporary practice, in which NIPT is offered to pregnant patients irrespective of maternal age or a priori risk, the predominant test indication was routine, patient-elected aneuploidy screening, followed by advanced maternal age, with abnormal ultrasound or serum screen findings representing a smaller proportion; test indications were not systematically coded in the laboratory information system, so exact proportions cannot be reported. Across the laboratory’s routine NIPT, the observed fetal fraction distribution was right-skewed, with a median fetal fraction of approximately 9.8% (range 3–34%; mode 7–8%), consistent with an unselected obstetric screening population. Of 11,318 consecutively accessioned pregnancies, 83 (0.73%) returned a not-reportable result (invalid, insufficient sample, or redraw without a result) and were excluded, yielding an evaluable cohort of 11,235, all with available diagnostic and/or clinical follow-ups (Figure 1). Each sample was collected in a single 10 mL Streck cell-free DNA blood collection tube (Streck, La Vista, NE, USA), couriered to the laboratory, and processed according to validated standard operating procedures. This laboratory reports four conditions—trisomy 21 (T21), trisomy 18 (T18), trisomy 13 (T13), and sex chromosome aneuploidies (SCA); other chromosomal findings are outside the reporting scope and were not included in the performance analysis. The de-identified dataset comprised maternal age, gestational age at collection, fetal fraction estimate, screening result category, and confirmatory/clinical follow-up outcome. This analysis used only de-identified clinical data; NIPT was performed under the ordering physicians’ standard clinical consent process (see the Institutional Review Board and Informed Consent Statements). Because this study was limited to de-identified data and did not involve any intervention or identifiable private information, it did not meet the definition of human subjects research requiring Institutional Review Board (IRB) review.

### 2.2. Assay and Sequencing Workflow

All samples were processed using the Illumina VeriSeq NIPT Solution v2 (Illumina, Inc., San Diego, CA, USA), a commercial whole-genome sequencing (WGS) assay implemented and analytically validated in house to meet Clinical Laboratory Improvement Amendments (CLIA) and College of American Pathologists (CAP) requirements [4,5]. The in-house analytical validation used 178 characterized clinical samples (278 data points, including replicates), with blinded, diagnostically confirmed specimens correlated against an independent reference laboratory and karyotype, and met predefined acceptance criteria (≥95% concordance) prior to clinical use (see Section 4.3). The assay uses genome-wide sequencing with a PCR-free library preparation; genome-wide coverage provides uniform representation across all chromosomes, supporting robust normalization and reliable fetal fraction estimation. Sequencing analysis and aneuploidy calling were performed using the VeriSeq NIPT Assay Software (part of the VeriSeq NIPT Solution v2; Illumina, Inc., San Diego, CA, USA). As noted above, only T21, T18, T13, and SCA were reported clinically.

This study was conducted using an automated, end-to-end workflow. All specimens were the routine clinical NIPT samples collected at the point of care in a single 10 mL Streck cfDNA blood collection tube prior to any diagnostic testing; no separate research samples were drawn. Automated liquid-handling systems (Hamilton Microlab STAR; Hamilton Company, Reno, NV, USA) performed plasma aspiration and cfDNA extraction. Extracted cfDNA underwent end repair and adapter ligation for library preparation, and paired-end sequencing was performed on the NextSeq 550Dx system (Illumina, Inc., San Diego, CA, USA). Bioinformatic analysis identified library fragments by index sequence, aligned paired-end reads to a human reference genome, estimated fetal fraction using fragment length distributions and genomic coordinates, applied a statistical model to detect genomic regions that were under- or over-represented after correcting for known biases, and generated an NIPT report with a fetal fraction estimate for each sample that passed quality control.

### 2.3. Confirmation of Outcomes and Follow-Up

When the assay returned a high-risk (aneuploidy) result, diagnostic confirmation by karyotype and/or chromosomal microarray following chorionic villus sampling or amniocentesis was performed when the patient consented to invasive testing. When a patient declined invasive diagnostic testing and elected to continue the pregnancy, the outcome was established by clinical work-up as directed by the treating clinician (for example, detailed ultrasound, serum screening, or postnatal or newborn assessment). True-positive and false-positive classifications were based on the best available confirmatory outcome. Low-risk results were classified as true-negative in the absence of a subsequently identified affected pregnancy on available clinical and newborn follow-up. Cases without sufficient follow-up to establish an outcome and aneuploidy-related positive signals not attributable to a reportable fetal condition—for example, those arising with a vanishing twin, molar pregnancy, or assisted reproduction—were not classified as confirmed reported-condition positives; 22 such cases across the cohort were reconciled out of the composite aneuploidy total accordingly.

### 2.4. Performance Evaluation

Clinical validity was assessed through five primary metrics: sensitivity (true-positive rate), specificity (true-negative rate), positive predictive value (PPV), negative predictive value (NPV), and observed prevalence within the cohort. Two-sided 95% confidence intervals were calculated using the Wilson score interval method. No dedicated statistical software package was used; confidence intervals (CIs; Wilson method) and the prevalence-adjusted positive predictive value model were computed manually using standard spreadsheet software. Performance was evaluated overall and stratified by maternal age (<35 and ≥35 years). For each condition, the expected PPV at general population prevalence was modeled as PPV = (p·Se)/[p·Se + (1 − p)·(1 − Sp)] to contextualize the observed values against the cohort’s observed prevalence. During the preparation of this manuscript, the authors used Claude (Opus 4.8; Anthropic, PBC, San Francisco, CA, USA) to assist with language editing, formatting, and verification of statistical calculations. All AI-assisted content was subsequently reviewed and edited by the authors, who take full responsibility for the content and integrity of the manuscript.

## 3. Results

The assay achieved a strong performance across all four reported conditions. The invalid/not-reportable rate was 0.73% (83 of 11,318 accessioned samples; Figure 1). This is at the low end of published first-pass NIPT failure rates, which generally range from approximately 1% to 5% and are typically higher for SNP-based assays that require a minimum fetal fraction [6,7,8]. In contemporaneous operational data, the first-pass repeat (redraw/re-extraction) rate was approximately 2%, and the invalidation (final no result) rate was approximately 0.9%, consistent with the study cohort; a low fetal fraction (below the assay’s ~3% threshold) was the predominant cause of repeats and invalidation, flagged samples were repeated on residual plasma when available, and roughly half were resolved on repeat. The laboratory’s turnaround time goal is a report within five business days of the specimen receipt, which was met in more than 95% of cases (98–100% in the most recent 12 months); the average business day turnaround (receipt to report) was approximately 2.7–3.0 business days. This laboratory metric is distinct from the overall patient/provider timeline, which also includes the collection, transport, and clinical review. The sensitivity (detection rate) was 100% for all conditions; specificity exceeded 99.9% for each individual condition and was 99.81% for any reported aneuploidy overall (reflecting the pooled false-positive count across the four conditions). The corresponding per-condition false-positive rates were 0.04% (T21), 0.03% (T18), 0.07% (T13), and 0.05% (SCA). No false-negative results were identified among cases with available follow-ups. The overall clinical performance is summarized in Table 1; performance stratified by maternal age is shown in Table 2 and Table 3; and a comparison with the published reference laboratory performance is shown in Table 4.

Among the 11,114 pregnancies with a low-risk (screen-negative) result, no false-negative result was identified on available follow-ups; a low-risk result therefore greatly reduced the likelihood of an affected fetus for the conditions screened, though it does not exclude other genetic conditions not evaluated by the assay. Of the 121 high-risk (screen-positive) results, 100 were confirmed to be affected across the four reported conditions (positive predictive value 82.6% for any reported aneuploidy); high-risk results were strongly correlated with the presence of abnormalities but, as with all screening tests, did not constitute a diagnosis.

The observed PPVs for T21 (91.8%; 95% CI 80.8–96.8%) and T18 (85.7%; 95% CI 65.4–95.0%) are consistent with published ranges [9,10,11,12]. No false-negative results were identified among cases with available follow-ups; a low-risk result therefore strongly favored an unaffected pregnancy for the conditions screened, although negative outcomes were not completely ascertained and NIPT does not exclude conditions outside the assay’s scope.

## 4. Discussion

These results demonstrate that a high-complexity clinical laboratory can achieve a level of clinical validity consistent with the benchmarks established by large-scale reference laboratories. The 100% sensitivity observed for trisomies 21, 18, and 13 aligns with high-performance metrics reported in meta-analyses involving more than 200,000 pregnancies [3]. The observed PPVs (T21 91.8%, T18 85.7%) are consistent with published ranges [9,10,11,12]. The cohort was ascertained in a routine, unselected obstetric screening population rather than a high-risk referral cohort; the predominant indication was patient-elected screening, followed by advanced maternal age, with abnormal serum or ultrasound findings representing a smaller proportion. The observed prevalence of each condition nonetheless exceeded livebirth-based general population estimates (for example, T21 0.40% versus a reported livebirth prevalence of approximately 0.14% [13]), as is expected for two reasons unrelated to high-risk selection: NIPT ascertains aneuploidy at the time of screening—earlier in gestation and therefore before the natural attrition that reduces the livebirth prevalence of autosomal trisomies relative to their prevalence at the time of screening [14]—and the cohort’s maternal age distribution skewed modestly older (28% were of advanced maternal age, ≥35 years), consistent with contemporary obstetric practice. Because the PPV rises with prevalence, the observed PPVs sit at the upper end of published ranges; comparisons with published data are therefore contextual and not adjusted for the case mix. Whole-genome sequencing analyzes cfDNA fragments across all chromosomes, which can improve robustness at low fetal fractions relative to targeted or SNP-based approaches [4,5]; the present single-assay, retrospective design does not permit a direct comparison of methods. These findings are concordant with recent large-scale and genome-wide NIPT cohorts [15,16].

### 4.1. Trisomy 13 Performance in the Context of Published Assay Data

Trisomy 13 warrants a specific comment because its PPV is strongly influenced by a low prevalence and by biological factors, such as confined placental mosaicism [17] and, less commonly, maternal copy number variants [18]; the placental, maternal, fetal, and technical origins of false-positive cell-free DNA results have recently been reviewed comprehensively [19]. The observed T13 PPV of 63.6% (95% CI 43.0–80.3%) is consistent with the published performance of the Illumina VeriSeq NIPT Solution v2, in which the modeled T13 PPV rises from approximately 50% at a prevalence of 0.10% to approximately 67% at a prevalence of 0.20% [4]. At the T13 prevalence observed in this cohort (0.12%), the corresponding assay-modeled PPV is approximately 53–55%; our observed value of 63.6% therefore sits at the upper end of the expected range. Applying the same prevalence-adjusted approach to the other reported conditions, the expected PPVs for the general population prevalence are approximately 80.0% (T21), 55.5% (T18), and 82.4% (SCA), versus observed values of 91.8%, 85.7%, and 79.3%; the higher observed PPVs for T21 and T18 reflect the cohort’s elevated prevalence, whereas the SCA value is close to the modeled expectation. Of the 23 SCA true-positives, 6 were confirmed by diagnostic cytogenetic testing (karyotype and/or chromosomal microarray) and 1 by postnatal evaluation; the remaining cases were classified as affected on the basis of concordant clinical findings (for example, multiple ultrasound anomalies or fetal demise) rather than cytogenetic confirmation, so the SCA positive predictive value should be interpreted with particular caution. These estimates are based on modest numbers of positive cases, and their wide confidence intervals should be interpreted with caution.

For sex chromosome aneuploidies, the observed PPV was 79.3% (95% CI 61.6–90.2%), consistent with published ranges [12,20]. Taken together, these findings indicate that the analytical and clinical performance achievable in an accredited local laboratory is consistent with that of established reference laboratories using the same class of technology.

### 4.2. Operational Considerations

Beyond diagnostic performance, performing NIPT within a local accredited clinical laboratory offers operational benefits that support integrated clinical care. The principal real-world contributions of this study are operational: an average business day turnaround of approximately 2.7–3.0 business days (receipt to report) with the five-business-day goal met in more than 95% of cases, a low invalid/not-reportable rate of 0.73%, and a prevalence-adjusted analysis that contextualizes the observed predictive values. Because testing was performed locally, a final report was available to the provider within an average of approximately three business days of receipt—compared with the additional transit and multi-day turnaround typical of send-out reference laboratory testing—shortening the interval to results, genetic counseling, and, when indicated, diagnostic testing. Results could be interpreted immediately within the context of the patient’s clinical situation, age, and family history. High-risk results could be managed promptly with coordinated diagnostic follow-up, and localized processing minimized the administrative and logistical delays inherent in send-out models, facilitating faster clinical intervention and counseling.

### 4.3. Implementation Considerations

The laboratory was established and analytically validated according to CAP standards within approximately six months. Implementation followed a staged process: the selection of a whole-genome sequencing assay and instrumentation (automated liquid handler and NextSeq 550Dx sequencer); laboratory information system integration; personnel training and competency assessment; and in-house analytical validation. The validation was performed against 178 characterized clinical samples (278 data points, including replicate testing and blinded, diagnostically confirmed abnormal specimens) correlated with an independent reference laboratory and karyotype and encompassed accuracy, precision (repeatability and reproducibility), sensitivity, specificity, fetal fraction concordance, and limit of detection. Predefined acceptance criteria required ≥95% concordance for each study, all of which were met, and quality control and proficiency testing procedures—including College of American Pathologists (CAP) proficiency testing—were established prior to a monitored clinical go-live phase under the direction of the CLIA laboratory director. Laboratories considering in-house NIPT should budget for this validation and quality assurance burden alongside the clinical and operational benefits.

### 4.4. Limitations

This study has several limitations. It is a single-laboratory, retrospective analysis, which may limit generalizability to other settings and populations. The cohort reflected routine, unselected prenatal screening rather than high-risk referral; nonetheless, because its maternal age distribution skewed modestly older and prevalence was ascertained at the time of screening, the observed prevalence and predictive values are not directly comparable to livebirth-based general population estimates. Although the sensitivity was 100% across conditions, the number of confirmed affected cases for individual aneuploidies—particularly T13 (*n* = 14) and SCA (*n* = 23)—is modest, producing wide confidence intervals; larger cohorts are needed to refine these estimates. Moreover, most SCA true-positives were confirmed by concordant clinical findings rather than cytogenetic testing (6 of 23 cytogenetically confirmed), so the SCA predictive values in particular should be regarded as provisional. Performance depends on the completeness of the diagnostic and clinical follow-up, and the incomplete ascertainment of outcomes (for example, low-risk pregnancies lost to follow-up) could cause increases in sensitivity and NPVs. Outcomes of the 83 not-reportable (no-result) cases were not ascertained; because no-result cfDNA screens are enriched for chromosomal abnormality [8], their exclusion is a potential source of ascertainment bias, and such patients warrant genetic counseling and a consideration of diagnostic testing [1]. The analysis was limited to the four conditions reported by this laboratory (T21, T18, T13, SCA); other chromosomal findings that a genome-wide assay can detect are outside the reporting scope and were not evaluated. Finally, as a screening test, NIPT cannot establish a diagnosis, and high-risk results must be confirmed by diagnostic testing.

## 5. Conclusions

This analysis of 11,235 pregnancies confirms that a clinical-grade WGS NIPT assay can be successfully implemented in a high-complexity clinical laboratory, achieving sensitivity and predictive values consistent with published ranges for established reference laboratories in a routine, unselected prenatal screening population. NIPT in the United States is performed by a spectrum of laboratories, from a few high-volume national reference laboratories to many smaller regional and local laboratories; our findings indicate that a local high-complexity laboratory can achieve a performance consistent with published benchmarks while offering faster turnaround and integrated counseling. NIPT remains a screening test; high-risk results require diagnostic confirmation, and no irreversible clinical decisions should be made on the basis of a screening result alone.

## Figures and Tables

**Table 1:** Overall clinical performance data (*N* = 11,235).

Target	Observed Prevalence	Sensitivity	Specificity	PPV	NPV *
Trisomy 21	0.40%	100%	99.96%	91.8%	100%
Trisomy 18	0.16%	100%	99.97%	85.7%	100%
Trisomy 13	0.12%	100%	99.93%	63.6%	100%
SCA (any)	0.20%	100%	99.95%	79.3%	100%
Any reported aneuploidy	0.89%	100%	99.81%	82.6%	100%

PPV, positive predictive value; NPV, negative predictive value; SCA, sex chromosome aneuploidy. CI, confidence interval. “Any reported aneuploidy” comprises the four reported conditions (T21, T18, T13, SCA); other chromosomal findings are outside the reporting scope. PPV 95% confidence intervals: T21 80.8–96.8, T18 65.4–95.0, T13 43.0–80.3, SCA 61.6–90.2, any reported aneuploidy 74.9–88.4; sensitivity 95% CI 92.1–100 (T21). * No false-negative results were identified among cases with available follow-up; because negative outcomes were not completely ascertained, NPV is an observed value that may be overestimated.

**Table 2:** Clinical performance in patients aged ≥35 years (*N* = 3118).

Target (≥35 y)	TP	FP	FN	TN	Total	Sens. (%)	Spec. (%)	Prev. (%)	PPV (%)	NPV (%)	Acc. (%)
T21	36	1	0	3081	3118	100	99.97	1.15	97.30	100	99.97
T18	14	1	0	3103	3118	100	99.97	0.45	93.33	100	99.97
T13	11	5	0	3102	3118	100	99.84	0.35	68.75	100	99.84
SCA (any)	6	3	0	3109	3118	100	99.90	0.19	66.67	100	99.90
Any reported aneuploidy	67	10	0	3041	3118	100	99.67	2.15	87.01	100	99.68

TP, true-positive; FP, false positive; FN, false-negative; TN, true negative; Sens., sensitivity; Spec., specificity; Prev., prevalence; Acc., accuracy.

**Table 3:** Clinical performance in patients aged <35 years (*N* = 8117).

Target (<35 y)	TP	FP	FN	TN	Total	Sens. (%)	Spec. (%)	Prev. (%)	PPV (%)	NPV (%)	Acc. (%)
T21	9	3	0	8105	8117	100	99.96	0.11	75.00	100	99.96
T18	4	2	0	8111	8117	100	99.98	0.05	66.67	100	99.98
T13	3	3	0	8111	8117	100	99.96	0.04	50.00	100	99.96
SCA (any)	17	3	0	8097	8117	100	99.96	0.21	85.00	100	99.96
Any reported aneuploidy	33	11	0	8073	8117	100	99.86	0.41	75.00	100	99.86

Abbreviations as in Table 2.

**Table 4:** Comparison of the laboratory’s clinical NIPT performance with published performance of reference laboratory platforms.

Study/Population	Technology	T21 Sens.	T21 PPV	T18 Sens.	T18 PPV	T13 PPV
This laboratory (unselected screening)	Whole-genome NGS	100%	91.8%	100%	85.7%	63.6%
Dar 2022, SMART [9] (low/high risk)	SNP-based NGS	NR	85.7/97.5%	NR	50.0/81.3%	62.5/83.3%
Norton 2015, NEXT [7] (average risk)	Targeted cfDNA	100%	80.9%	90%	90.0%	50.0%
Strom 2017 [10] (clinical laboratory)	Massively parallel NGS	NR	98%	NR	92%	69%

Sens., sensitivity; PPV, positive predictive value; NGS, next-generation sequencing; SNP, single-nucleotide polymorphism; NR, not reported for individual conditions in a directly comparable form. PPVs are the values reported in each cited primary study [7,9,10]. The SMART study [9] reported separate low-risk/high-risk cohorts (shown as low-risk/high-risk); the average-risk NEXT cohort [7] is the most comparable to the present unselected screening population. Sensitivity for all reported conditions was 100% in the present study (Table 1). Values are provided for context and are not head-to-head measurements; because PPV depends on the prevalence and case mix of each study population, comparisons are not adjusted for these differences.

## Data Availability

The data presented in this study are available on request from the corresponding author. The data are not publicly available due to patient privacy restrictions.

## Abbreviations

The following abbreviations are used in this manuscript:

NIPT	non-invasive prenatal testing
cfDNA	cell-free DNA
WGS	whole-genome sequencing
NGS	next-generation sequencing
PPV	positive predictive value
NPV	negative predictive value
SCA	sex chromosome aneuploidy
T21/T18/T13	trisomy 21/18/13
CI	confidence interval
CLIA	Clinical Laboratory Improvement Amendments
CAP	College of American Pathologists
ACOG	American College of Obstetricians and Gynecologists
ACMG	American College of Medical Genetics and Genomics
CVS	chorionic villus sampling
